# Supplementary material for: EnzML: multi-label prediction of enzyme classes using InterPro signatures
Source: BMC Bioinformatics. 2012 Apr 25;13:61. doi: 10.1186/1471-2105-13-61 (PMC3483700; doi:10.1186/1471-2105-13-61)
Supplement: Addtional file 5 — The Java code to format the data files, evaluate and predict. The file enzml_java_code.tar.gz contains the Java code used to format database data to ARFF and XML formats, to execute cross and train-test (jackknife) evaluations and to record evaluation results to database. More information is included in the readme.txt file and the Javadoc files. The code can be used with a MySQL database. To use a different database software, other JDBC drivers might be required. [file 1471-2105-13-61-S5.gz › java_code/enzml2011/doc/uk/ac/ed/inf/enzml/test/dataharness/PathTest.html]

PathTest


---


|  |  |  |
| --- | --- | --- |
| **Overview** \| **Package** \| **Class** \| **Use** \| **Tree** \| **Deprecated** \| **Index** \| **Help** | |  |
| **PREV CLASS**   **NEXT CLASS** | **FRAMES**    **NO FRAMES**     **All Classes** |  |
| SUMMARY: NESTED \| FIELD \| CONSTR \| METHOD | DETAIL: FIELD \| CONSTR \| METHOD |  |


---


## uk.ac.ed.inf.enzml.test.dataharness Class PathTest

```
java.lang.Object
  uk.ac.ed.inf.enzml.test.dataharness.PathTest
```

---

``` public class PathTest extends java.lang.Object ```

---

| **Field Summary** | |
| --- | --- |
| `static java.lang.String` | `FILTER_OLD_TEST_SET` |
| `static java.lang.String` | `FILTER_TRAIN_SET` |
| `static java.lang.String` | `TEST_ARFF_DB_PROPS` |
| `static java.lang.String` | `TEST_ARFF_FILES_PATH` |
| `static java.lang.String` | `TEST_ARFF_PROPS_1` |
| `static java.lang.String` | `TEST_ARFF_PROPS_2` |
| `static java.lang.String` | `TEST_ARFF_PROPS_NO_XML` |
| `static java.lang.String` | `TEST_ARFF_PROPS_PATH` |
| `static java.lang.String` | `TEST_BASE_PATH` |
| `static java.lang.String` | `TEST_DB_CONN_PROPERTIES` |
| `static java.lang.String` | `TEST_MACHINE_LEARNING_PROPS` |
| `static java.lang.String` | `TEST_ML_PROPS_PATH` |
| `static java.lang.String` | `TEST_MODEL_FOLDER` |
| `static java.lang.String` | `TEST_PREDICTIONS_FOLDER` |
| `static java.lang.String` | `TEST_PROPS_PATH` |
| `static java.lang.String` | `TEST_RESULTS_FOLDER` |


**Constructor Summary**

`PathTest()`


**Method Summary**

**Methods inherited from class java.lang.Object**

`equals, getClass, hashCode, notify, notifyAll, toString, wait, wait, wait`

**Field Detail**

### TEST\_BASE\_PATH

```
public static final java.lang.String TEST_BASE_PATH
```

**See Also:**: Constant Field Values

---


### TEST\_PREDICTIONS\_FOLDER

```
public static final java.lang.String TEST_PREDICTIONS_FOLDER
```

**See Also:**: Constant Field Values

---


### TEST\_PROPS\_PATH

```
public static final java.lang.String TEST_PROPS_PATH
```

**See Also:**: Constant Field Values

---


### TEST\_ARFF\_FILES\_PATH

```
public static final java.lang.String TEST_ARFF_FILES_PATH
```

**See Also:**: Constant Field Values

---


### TEST\_ARFF\_PROPS\_PATH

```
public static final java.lang.String TEST_ARFF_PROPS_PATH
```

**See Also:**: Constant Field Values

---


### TEST\_ML\_PROPS\_PATH

```
public static final java.lang.String TEST_ML_PROPS_PATH
```

**See Also:**: Constant Field Values

---


### TEST\_ARFF\_PROPS\_NO\_XML

```
public static final java.lang.String TEST_ARFF_PROPS_NO_XML
```

**See Also:**: Constant Field Values

---


### TEST\_MACHINE\_LEARNING\_PROPS

```
public static final java.lang.String TEST_MACHINE_LEARNING_PROPS
```

**See Also:**: Constant Field Values

---


### TEST\_ARFF\_PROPS\_1

```
public static final java.lang.String TEST_ARFF_PROPS_1
```

**See Also:**: Constant Field Values

---


### TEST\_ARFF\_PROPS\_2

```
public static final java.lang.String TEST_ARFF_PROPS_2
```

**See Also:**: Constant Field Values

---


### TEST\_ARFF\_DB\_PROPS

```
public static final java.lang.String TEST_ARFF_DB_PROPS
```

**See Also:**: Constant Field Values

---


### TEST\_DB\_CONN\_PROPERTIES

```
public static final java.lang.String TEST_DB_CONN_PROPERTIES
```

**See Also:**: Constant Field Values

---


### TEST\_RESULTS\_FOLDER

```
public static final java.lang.String TEST_RESULTS_FOLDER
```

**See Also:**: Constant Field Values

---


### TEST\_MODEL\_FOLDER

```
public static final java.lang.String TEST_MODEL_FOLDER
```

**See Also:**: Constant Field Values

---


### FILTER\_OLD\_TEST\_SET

```
public static final java.lang.String FILTER_OLD_TEST_SET
```

**See Also:**: Constant Field Values

---


### FILTER\_TRAIN\_SET

```
public static final java.lang.String FILTER_TRAIN_SET
```

**See Also:**: Constant Field Values


**Constructor Detail**

### PathTest

```
public PathTest()
```


---


|  |  |  |
| --- | --- | --- |
| **Overview** \| **Package** \| **Class** \| **Use** \| **Tree** \| **Deprecated** \| **Index** \| **Help** | |  |
| **PREV CLASS**   **NEXT CLASS** | **FRAMES**    **NO FRAMES**     **All Classes** |  |
| SUMMARY: NESTED \| FIELD \| CONSTR \| METHOD | DETAIL: FIELD \| CONSTR \| METHOD |  |


---
